# 2-Deoxyglucose Reverses the Promoting Effect of Insulin on Colorectal Cancer Cells *In Vitro*

**DOI:** 10.1371/journal.pone.0151115

**Published:** 2016-03-03

**Authors:** Dongsheng Zhang, Qiang Fei, Juan Li, Chuan Zhang, Ye Sun, Chunyan Zhu, Fengzhen Wang, Yueming Sun

**Affiliations:** 1 Department of Colorectal Surgery, The First Affiliated Hospital of Nanjing Medical University, Nanjing, Jiangsu, China; 2 The First School of Clinical Medicine, Nanjing Medical University, Nanjing, Jiangsu, China; 3 Department of Pharmaceutics, China Pharmaceutical University, Nanjing, Jiangsu, China; Southern Illinois University School of Medicine, UNITED STATES

## Abstract

An increased risk of colorectal cancer is related to the development of metabolic syndromes including hyperglycemia, and hyperinsulinemia. The high circulatory levels of glucose and/or insulin or the application of exogenous insulin may promote carcinogenesis, cancer progression and metastasis, which can be attributed to the Warburg effect or aerobic glycolysis. We attempted to resolve these existing questions by applying the glucose analog 2-deoxyglucose (2DG). According to the *in vitro* studies we performed, the glycolysis of colorectal cancer cells could be interrupted by 2DG as it decreased the cellular productions of ATP and lactate. In addition, 2DG induced apoptosis and cell cycle arrest, and inhibited proliferation, migration and invasion of these cells. Since insulin can stimulate the cellular uptake of hexose, including 2DG, the combination of 2DG and insulin improved the cytotoxicity of 2DG and meanwhile overcame the cancer-promoting effects of insulin. This *in vitro* study provided a viewpoint of 2DG as a potential therapeutic agent against colorectal cancer, especially for patients with concomitant hyperinsulinemia or treated with exogenous insulin.

## Introduction

Colorectal cancer (CRC) is known to be strongly associated with a western lifestyle. The incidence rises rapidly over the last century in parallel with the booming economic development [1].Given the increased morbidity of metabolic syndromes, many studies have been conducted to investigate their connection with CRC. Evidences suggest that type 2 diabetes mellitus (DM), insulin resistance, hyperinsulinemia are independent risk factors for colorectal cancer [2,3].

**Fig 1:**
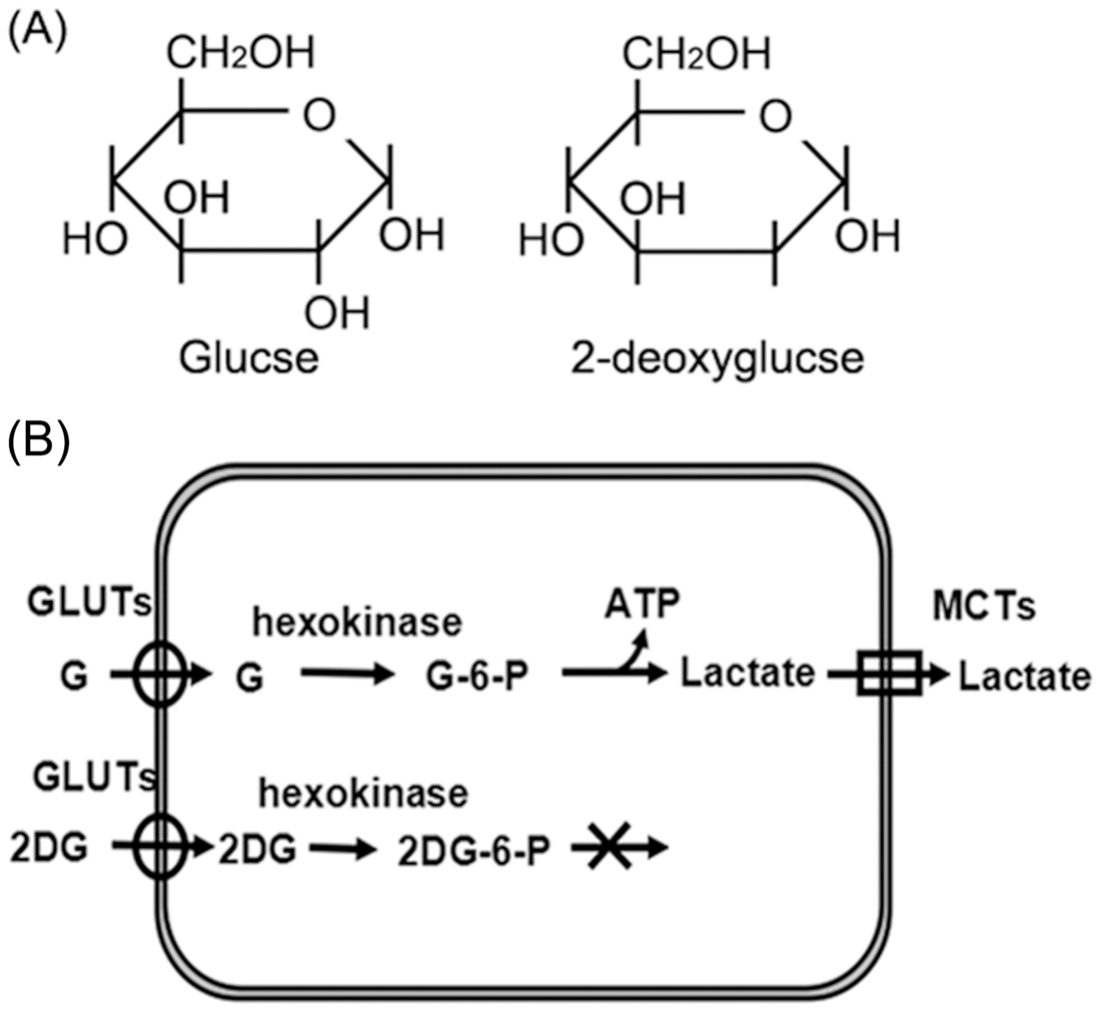
Molecular structure of 2-deoxyglucose and its inhibition of glycolysis. (A) Molecular structure of glucose and 2-deoxyglucose. (B) Because of structural resemblance with glucose, 2-deoxyglucose enters the cell via gluts, leading to the interruption of glycolysis with decreased productions of ATP and lactate[6–8]. G:glucose. 2DG: 2-deoxyglucose.

Type 2 DM is characterized by hyperglycemia resulting from the combination of insulin resistance and a relative lack of insulin. High circulating glucose level is likely to favor the development of cancer. The main reason is that most cancer cells predominantly rely on aerobic glycolysis to generate the energy needed for cellular processes, a phenomenon known as the Warburg effect [4]. Apart from being the main energy source, glucose is used as a major carbon source for anabolic reactions [5].This characteristic has been taken advantage of to image cancer in clinics by applying 2-(18F)-fluoro-2-deoxy-D-glucose (FDG) in positron emission tomography (PET). Targeting the glucose metabolism has become a potential strategy against cancer. One of the most promising glycolytic inhibitors is 2-deoxyglucose (2DG) [6–8]. 2DG is a synthetic glucose analog which has the C-2 hydroxyl group replaced by hydrogen (Fig 1A). After entering the cell via glucose transporters (Gluts), 2DG is converted by hexokinase to form phosphorylated 2DG which accumulates in the cell, leading to the non-competitive inhibition of hexokinase, decreased productions of ATP and lactate, and eventually cell growth inhibition and cell death (Fig 1B)[6–8].

In addition to the effects of hyperglycemia, insulin resistance and compensatory hyperinsulinemia are also important contributors to the development and progression of several neoplasms [9]. Insulin has been confirmed to be capable of stimulating glucose uptake in many cancer cells [10], which may promote the Warburg effect. Insulin can also exert mitogenic and antiapoptotic effects [11–13]. Besides, insulin can amplify the bioavailability of insulin like growth factor-1 (IGF-1) [14–16]. Patients with concomitant colorectal cancer and type 2 DM who may also use insulin are facing the potential threat that insulin may promote cancer progression. Studies with animal models have already confirmed the assumption [17]. Although currently the relationship between insulin or insulin resistance and colorectal cancer is not explicit, no one can ignore the potential effects of insulin at various stages of carcinogenesis.

Understanding the glucose metabolism and the function of insulin in colorectal cancer cells will promote the development of some novel approaches for its prevention and treatment. This *in vitro* study aims to determine the anticancer effects of 2DG and the effects of insulin on colorectal cancer cell lines. In addition, this study investigated the possibility of insulin in enhancing the anticancer efficiency of 2DG.

## Materials and Methods

### Cell culture

Two colorectal cancer cell lines (HCT116, LoVo) were obtained from the Cell Bank of Type Culture Collection of the Chinese Academy of Sciences (Shanghai, China) and cultured in high-glucose Dulbecco’s modified Eagle’s medium (DMEM) (4.5g/l glucose) containing 10% fetal bovine serum (FBS), 1% penicillin-streptomycin, in a 5% CO_2_ humidified incubator at 37°C.

### Chemicals

2DG and insulin from bovine pancreas were purchased from Sigma (St. Louis, MO). Drugs were dissolved in complete culture medium. Solutions were filter sterilized using 0.22-μm syringe-filter units (Beyotime Biotechnology, Shanghai, China).

### Cell proliferation assay

Cell Counting Kit-8 (CCK8) assay for cellular proliferation was performed according to the manufacturer’s instruction (Beyotime Biotechnology, Shanghai, China). Cells were treated with 2DG and/or insulin for 24, 48 or 72h. Then culture media were replaced with fresh media supplemented with cell proliferation reagent. After 2h incubation, measurements were performed using a 96-well spectrophotometric plate reader (Sunrise-Basic Tecan, Austria) with the absorbance wavelength at 450nm. Effect of insulin on cellular proliferation was evaluated and an appropriate insulin concentration was determined for succeeding studies.

### Flow cytometry in analysis of apoptosis and cell cycle

Flow cytometry in analysis of apoptosis was performed using a double staining method with Annexin-V-FITC and propidium iodide (PI). The assay was carried out using Apoptosis Kit (KeyGen Biotech. Co. Ltd., Nanjing, China) according to the manufacturer’s instruction. Cells were cultured with 2DG and/or insulin for 24h and then trypsinized without EDTA, washed with PBS, stained with Annexin V-FITC and PI and analyzed by flow cytometry (Becton-Dickinson, San Jose, CA). Data were processed by Kaluza flow cytometry analysis software 1.2 (Beckman Coulter).

For cell cycle analysis, Cell Cycle Analysis Kit from Beyotime Biotechnology (Shanghai, China) was used based on the manufacturer’s instruction. Cells were collected, washed with PBS, fixed in 75% cold ethanol and incubated overnight at 4°C. Then cells were washed and re-suspended in staining solution containing PI and RNAseA and incubated for 30 minutes at 37°C before flow cytometry analysis. Cell cycle fractions were quantified with WinCycle software (Phoenix Flow Systems, San Diego, CA).

### Cell migration and invasion assay

The migration and invasion of colorectal cancer cells were assessed using Transwell Permeable Supports (Corning Incorporated, Corning, NY, MA) with filters of 8.0 μm pore size, 6.5 mm diameter. Cancer cell suspensions in DMEM (5×10E5 cells/ml, 100μl) supplemented with or without drug were added to the upper compartment of the chamber and 600μL DMEM containing 10% FBS was added to the lower compartment. After 24h incubation, the filters were harvested and immersed in crystal violet. Cells on the upper surface of the filter were wiped away with cotton swabs. The number of cells that passed through the filter was counted in five fields under a microscope. For the *in vitro* invasion assay, Matrigel (BD Biosciences) was added to the upper surface (50μl/cm^2^) to form a matrix barrier and the inoculated cell concentration was 1×10E6 cells/ml.

### ATP content analyses

The intracellular ATP content was determined using a luciferin-luciferase-based method. Cells were incubated with 2DG and/or insulin for 24 h. Then the cells were trypsinized, counted and processed using ATP Assay Kit (Beyotime Biotechnology, Shanghai, China) and measurements were performed using a luminometer (GloMaxTM 20/20, Promega Corporation, Madison, WI). The ATP production was normalized to cell number and expressed as percent of normalized ATP values found in control cells.

### Lactate production analyses

Lactate production analyses were performed using lactate assay kit (Beyotime Biotechnology, Shanghai, China). After 24h treatment with 2DG and/or insulin, cells were scraped off with a cell scraper and cell numbers were counted. Then the suspensions were sonicated on ice for 3 cycles. Each cycle consisted of 5s sonication with a 100W power output from a Qsonica ultrasonic cell disruptor (Newtown, CT) followed by 30s incubation. The sonicated samples were assayed to determine the whole lactate levels following the manufacturer’s instruction. Extracellular lactate levels were also determined as the culture media were collected and tested. Measurements were performed using a Tecan spectrophotometric plate reader with the absorbance wavelength at 540 nm. The lactate productions were normalized to cell number and expressed as percent of the control.

### Protein extraction and Western blotting

Cells were treated with 20uU insulin and/or 5mM 2DG for 24h and then were lysed with radioimmune precipitation assay (RIPA) buffer (Beyotime biotechnology, Shanghai, China) supplemented with 1% phenylmethanesulphonyl fluoride (PMSF) (Beyotime biotechnology). Protein concentrations were determined using a BCA Protein Assay Kit (Beyotime biotechnology). Samples were mixed with 5×SDS-PAGE sample loading buffer. Equal amounts of whole protein extracts were electrophoresed through a polyacrylamide gel and transferred to a polyvinylidene difluoride (PVDF) membrane (Millipore, Billerica, MA) by Wet Electrophoretic Transfer. Membranes were blocked in 5% skim milk in Tris-buffered saline plus 0.1% Tween 20 (TBS-T) and then incubated overnight with indicated primary antibodies against Phospho-AMPKα (Thr172) (Cell Signaling Technology), AMPKα (Cell Signaling Technology), SLC16A3/MCT4 (Abcam), LC3A/B(Cell Signaling Technology), GAPDH(Cell Signaling Technology), Mcl-1(Cell Signaling Technology), Survivin(Cell Signaling Technology), MMP2(Cell Signaling Technology), p62(Cell Signaling Technology). The blots were then washed and incubated with horseradish peroxidase-conjugated secondary antibodies (Jackson ImmunoResearch Laboratories Inc., USA) and visualized with Immobilon™ Western Chemiluminescent HRP Substrate (Millipore Corp, USA) using the FluorChem E system (ProteinSimple, Santa Clara, CA).

### Statistical Analysis

Data were expressed as mean ± SD from at least three independent experiments. Statistical analysis was performed based on the Student’s t-test. A P-value of less than 0.05 was considered statistically significant. The statistical operations were performed using Statistical Package for Social Science version 19 software (SPSS, Chicago, IL).

## Results

### Insulin enhanced the antiproliferation and apoptosis induction of 2DG *in vitro*

**Fig 2:**
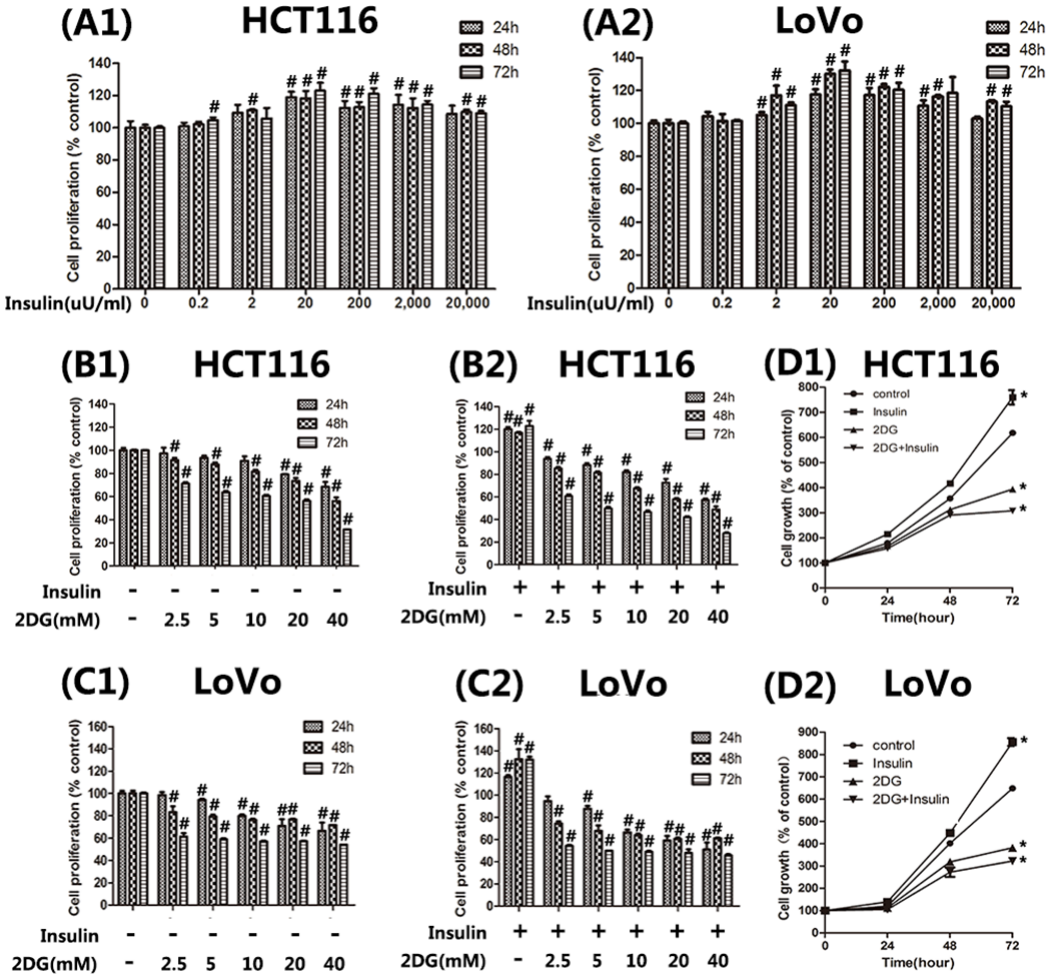
Cellular proliferation assay. Percentages of viable cells at different time points (24,48,72h) were shown. The data are presented as the mean ± SD of the results obtained from three independent experiments. (A) Cellular proliferation in response to ingredient concentrations of insulin. (B and C) HCT116 and LoVo colorectal cancer cells were treated with 2DG at indicated concentrations with or without the presence of insulin (20μU/ml). (D) Cells were treated for 72h with 5mM 2DG and/or 20μU/ml insulin. Cell growth curves were constructed. *, significant differences versus the controls (P < 0.05). #, significant differences versus control at the same time (P < 0.05).

In the first series of experiments, we examined whether 2DG possessed any antiproliferative effect in colorectal cancer cell lines. Cell proliferation assay was performed every 24 hours for 72 hours. Data showed that 2DG exerted a significant inhibitory effect on the proliferation of both cells compared with control (Fig 2B1 and 2C1). The inhibitory effect was dose- and time- dependent. In contrast, insulin generally exhibited a modest promotion on cell proliferation (Fig 2A), which did not increase in parallel with the increase in insulin concentration. 20μU/ml insulin close to the upper limit of normal physiological level in the fasting state was selected for succeeding studies. The combination of 2DG and insulin (20μU/ml) resulted in a significantly greater suppression of cellular proliferation compared with 2DG treatment alone (Fig 2B and 2C).

**Fig 3:**
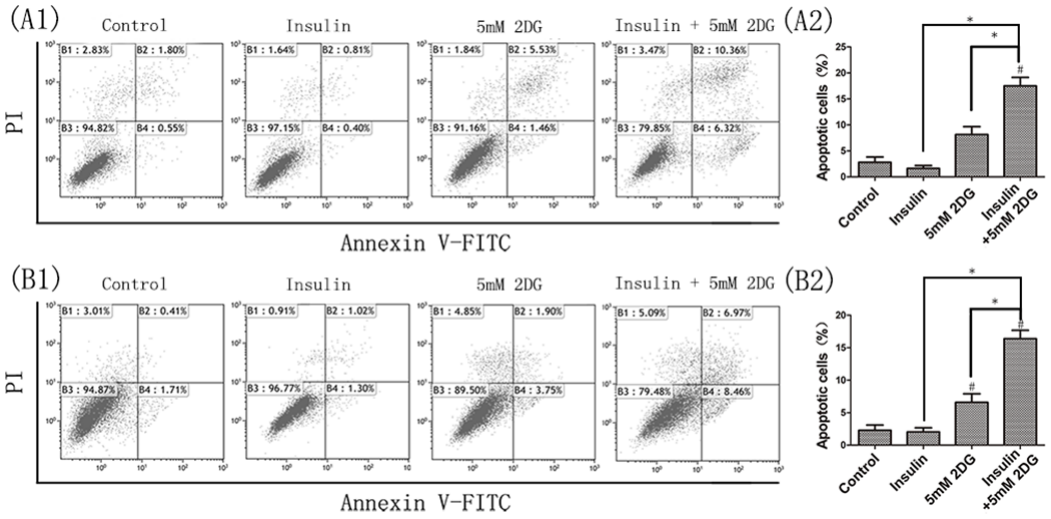
Flow cytometry in analysis of apoptosis. Flow cytometry analysis of cell apoptosis was detected by PI and Annexin V-FITC staining (A: HCT116, B: LoVo). Cells were treated with 5mM 2DG and/or 20μU/m insulin. Experiment was performed in triplicate. Representative images were shown. *, P < 0.05. #, significant differences versus the controls (P < 0.05).

**Fig 4:**
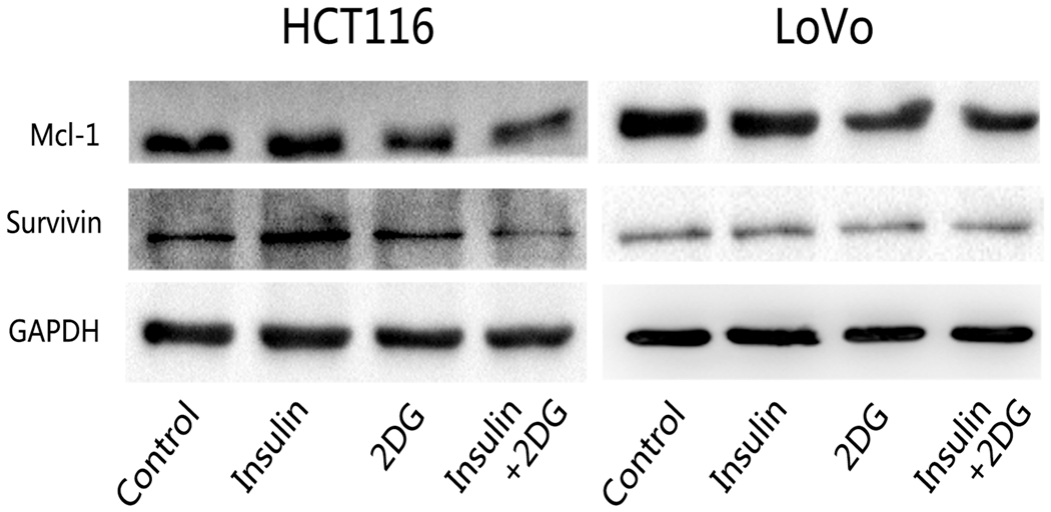
Western blots of Mcl-1 and survivin. Western blot analysis of Mcl-1 and survivin. GAPDH was used as a loading control.

Cell apoptosis analysis by flow cytometry showed a mild increase in cancer cell apoptosis following 2DG treatment *in vitro* (Fig 3). The proportions of annexin V-positive/PI-negative cells, indicating early apoptotic cells, and Annexin V-positive/PI-positive cells, indicating late apoptotic or necrotic cells, increased in a dose-dependent manner (data not shown) under the effect of 2DG. Western blot analysis showed that 2DG inhibited the expressions of survival proteins Mcl-1 and survivin (Fig 4). Although not statistically significant, insulin tends to suppress cancer cell apoptosis. When insulin was concomitantly administered, the apoptotic cells were further increased compared with 2DG monotherapy (Fig 3). The results of cellular proliferation and apoptosis assays suggested that insulin promoted the anticancer effects of 2DG and that 2DG reversed the cancer promoting effects of insulin.

### 2DG induced G1 cell cycle arrest *in vitro*

**Fig 5:**
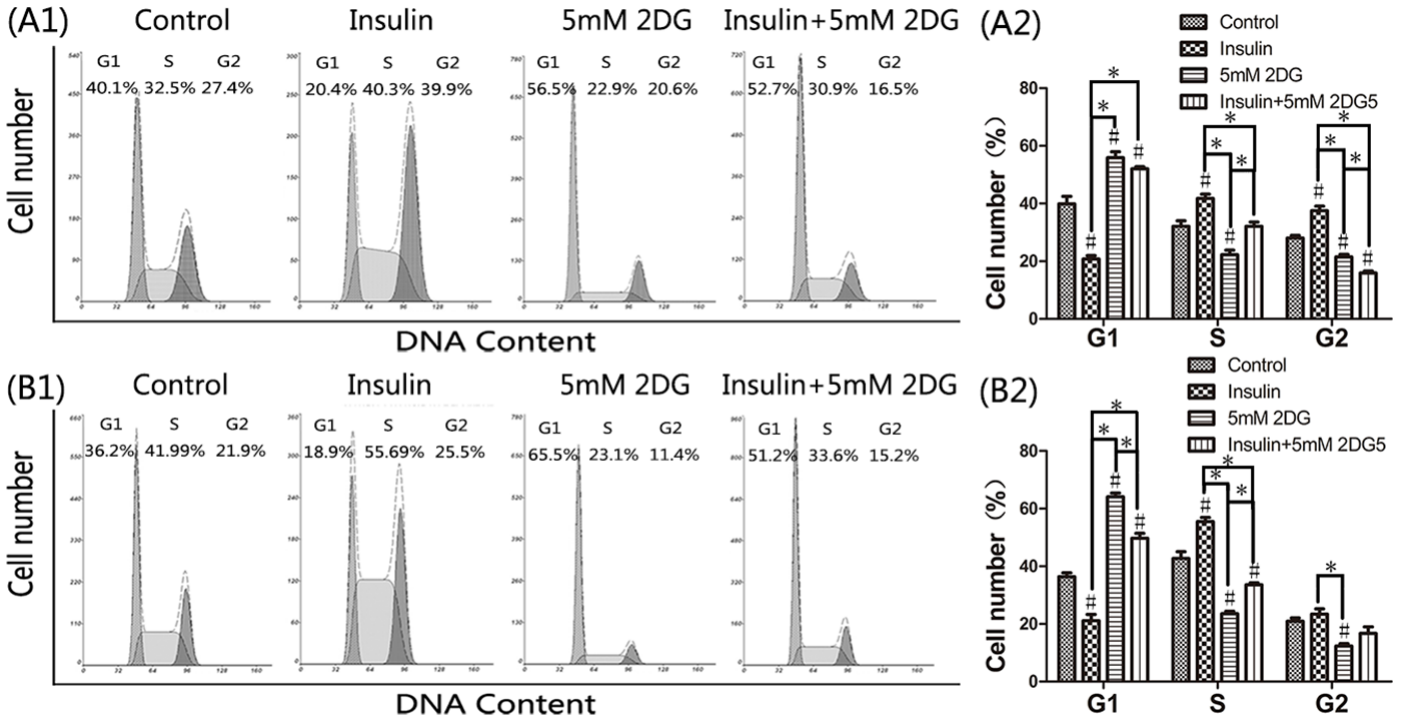
Flow cytometry in analysis cell cycle. Cell cycle distribution of cancer cells treated under varying conditions (A: HCT116, B: LoVo). Experiment was performed in triplicate. Representative images were shown. *, P < 0.05. #, significant differences versus the controls (P < 0.05).

Rapidly proliferating cancer cells are addicted to increased amounts of glucose not only for energy production but also for the biosynthesis of nucleic acids, proteins, and lipids [18]. The interference with glucose metabolism might arrest or delay cell cycle progression. Flow cytometry analysis of cell cycle distribution showed that incubation of cells with 2DG resulted in an increased percentage of cells in G1 phase (Fig 5). Conversely, insulin induced a sharp increase of cells in S and G2 phases. However, the G1 cell cycle arrest by 2DG was attenuated but not promoted by insulin.

### Combining 2DG with insulin markedly suppressed cancer cell migration and invasion *in vitro*

**Fig 6:**
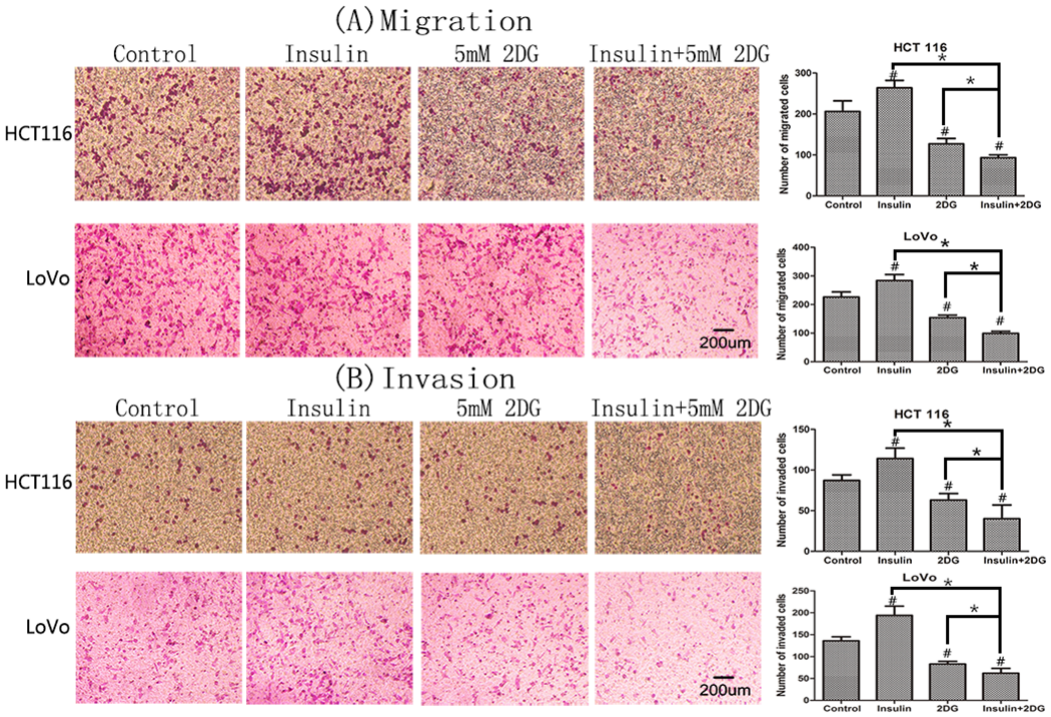
Cancer cell migration and invasion. In the migration and invasion analyses of cancer cells, five randomly selected fields were analyzed. Representative images are shown. The number of migrated and invaded cells were presented. Data were represented as mean ± SD. #, significant differences compared with controls (P < 0.05). * P < 0.05.

**Fig 7:**
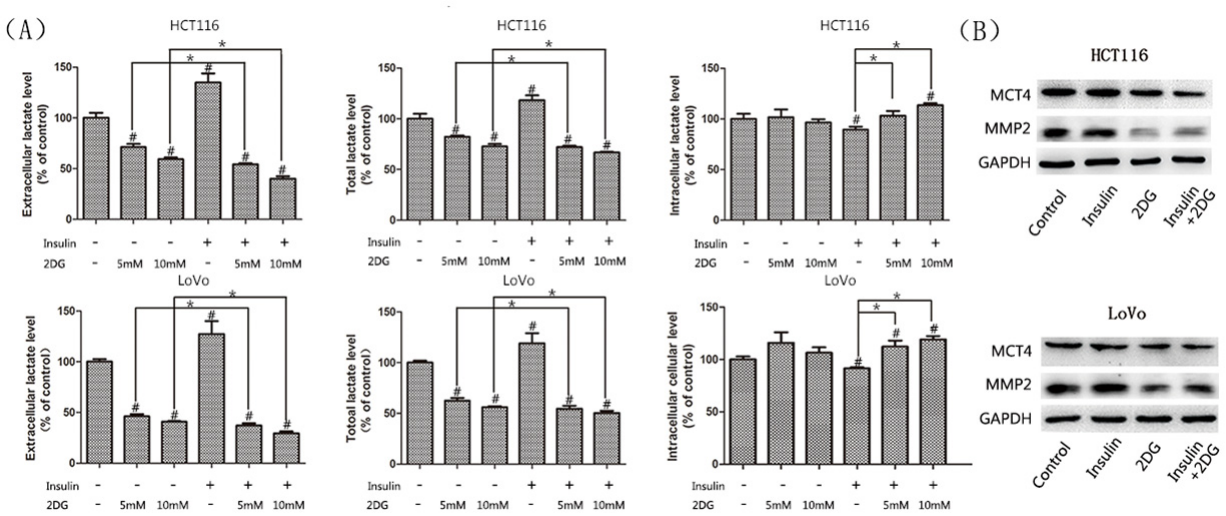
Lactate content analysis and western blots of MCT4 and MMP2. (A)The total and extracellular lactate levels were shown for cells treated with 2DG and/or insulin for 24h. The intracellular lactate was determined as the difference between those of the total and extracellular. #, significant differences compared with controls (P < 0.05). * P < 0.05. (B)Western blot analysis of SLC16A3/MCT4, MMP2. GAPDH was used as a loading control.

To determine whether 2DG could attenuate metastatic potential, Transwell Permeable Supports were utilized with and without Matrigel coating to assess invasion and migration, respectively. In comparison with the control group, invasion and migration were significantly inhibited in both cells treated with 2DG (Fig 6). Conversely, insulin significantly enhanced cancer cell migration and invasion. The numbers of migrated and invaded cells were respectively 1.28 folds and 1.32 folds of the controls in HCT 116, 1.25 folds and 1.42 folds in LoVo. Compared with 2DG monotherapy, the migration and invasion in both cells were further depressed by the combination of insulin and 2DG. The numbers of migrated and invaded cells dropped by over 50% of those in control groups. Western blot analysis showed that insulin promoted the expression of MMP2 protein—a key member among the matrix metalloproteinases, which might to some extent explain the invasion promoting effect of insulin. In contrast, 2DG inhibited the expression of MMP2 (Fig 7B).

### Combining 2DG with insulin led to marked decreases in ATP and lactate production *in vitro*

**Fig 8:**
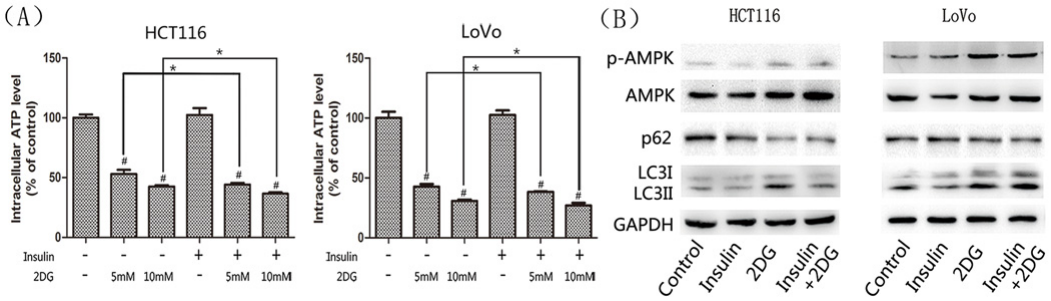
ATP content analysis and western blots of AMPK, p62 and LC3. (A) The ATP levels following the treatment of 2DG and/or insulin(20μU/ml) for 24h in colorectal cancer cells. ATP levels were normalized to untreated control sample. Data are mean ± SD of 3 independent experiments. #, significant differences compared with controls (P < 0.05). * P < 0.05. (B)Western blot analysis of Phospho-AMPKα (Thr172) representing active AMPK, AMPKα representing total AMPK, p62, LC3A/B. GAPDH was used as a loading control.

On the basis of the anticancer effects, we then investigated the effect of 2DG on glycolysis *in vitro*. Rapidly proliferating cancer cells are characterized by elevated glycolysis, therefore we hypothesized that 2DG would lead to glycolysis suppression due to its nonmetabolizable character and inhibition of some glycolysis enzymes. ATP and lactate levels in response to 2DG were analyzed. Significant decreases in total intracellular ATP levels were observed following 2DG administration (Fig 8A). Incubation of HCT116 and LoVo cells with 2DG at 5mM for 24 h led to ATP levels at 53% and 42% of the levels in untreated cells, respectively. The presence of insulin did not affect the ATP production. When the two drugs were applied together, the ATP contents were further decreased. As a consequence of ATP deprivation, 2DG induced the upregulation and activation of AMP-activated protein kinase (AMPK) as demonstrated by western blot analysis (Fig 8B). 2DG also induced the upregulation of LC3I and the conversion from LC3I to LC3II (Fig 8B), which suggested the upregulation of autophagy. Another marker of autophagy - p62- decreases with the elevation of autophagy as it can be degraded in this process [19]. As shown in Fig 8B, 2DG lowered the p62 level. 2DG structurally resembles glucose but it cannot be metabolized to generate energy thus mimicking the effect of glucose deprivation and activating autophagy.

As to the determination of lactate production *in vitro*, cell suspensions were sonicated and analyzed for the total lactate levels (Fig 7A). The difference between the total and extracellular lactate levels might indirectly indicate the intracellular lactate level. Results showed that both the total and extracellular lactate levels were increased by insulin compared with control, while surprisingly the intracellular lactate levels were slightly decreased in both cells. We hypothesized that lactate might be consumed by these normoxic cells for anabolism or exported much more greatly under the influence of insulin. As expected, 2DG significantly decreased lactate production. The combination of the two agents led to a further decreased lactate level while the intracellular lactate seemed to be mildly increased. Monocarboxylate transporter 4 (MCT4), involved in the extrusion of lactate, was significantly downregulated by the combination as shown by western blot (Fig 7B), which might explain the increase in intracellular lactate to some extent.

## Discussion

To Type 2 DM patients, hyperglycemia may increase cancer risk, which to some extent can be attributed to the Warburg effect—a phenomenon that most cancer cells predominantly produce energy by glycolysis rather than oxidative phosphorylation followed by an elevated secretion of lactic acid, even in the condition of sufficient oxygen tension [5]. Hyperinsulinemia may also be a potential contributor to cancer as the metabolic effect of insulin may promote the Warburg effect. A high (supraphysiological) dose of exogenous insulin is often required in the treatment of diabetes to achieve normoglycemia. While improving metabolic control, elevated insulin level may increase cancer risk by dose-dependent effects on cellular differentiation, growth, proliferation, migration and invasion [20]. For diabetes patients with cancer, the development of insulin resistance is reported to be combined with frequent insulin receptor overexpression and increased insulin activity in cancer cells [12]. Insulin can also hinder the efficacy of many chemotherapeutic drugs[21–23]. Although it still remains a question as to whether, or to what extent, exogenous insulin therapy causes cancer progression in patients, no one can overlook the potential threat of insulin which has been confirmed by *in vivo* animal studies [11].

As to colorectal cancer, diabetes has been suggested as a key contributor to its development [11]. Epidemiological studies observed a positive correlation between fasting hyperglycemia (≥ 6.1 mmol/l) and colorectal cancer incidence. Despite significant advances in surgical and medical treatment, colorectal cancer remains a highly prevalent and lethal malignancy in developed countries. Based on the above standpoints, we suggest that targeting the glucose metabolism in treating colorectal cancer is promising. Although, many cancer cells are sensitive and vulnerable to glucose deprivation [24]. This strategy is impracticable in mammals because the process of gluconeogenesis, mainly taking place in the liver, providing a source of glucose from non-carbohydrate carbon substrates, such as lactate, glycerol. Thus, it is achievable and sustainable to mimic glucose deprivation *in vivo* through inhibition of glucose metabolism by the application of pharmacological agents such as 2DG. Once transported into cells via Gluts, 2DG is phosphorylated by hexokinase to form 2DG-6-phosphate which cannot be further metabolized via glycolysis but rather accumulates and noncompetitively inhibits hexokinase and competitively inhibits phosphoglucose isomerase[18,25,26]. Therefore, normal glucose metabolism is disturbed, leading to energy deprivation and eventually cell death. Herein, we proposed 2DG as a potential candidate against colorectal cancer. Especially, when exogenous insulin is administered or circulatory hyperglycemia is combined, the efficiency of 2DG may be further improved since insulin can promote cellular uptake of hexose.

Our *in vitro* study conducted functional assays of colorectal cancer cells such as cell proliferation, apoptosis, migration, and invasion. Glycolysis was analyzed by the productions of ATP and lactate. 2DG and insulin were applied to treat these cells either alone or in combination. The results showed that 2DG had inhibitory effects on cancer cell while insulin had direct promoting effects. When insulin was combined, the anticancer effects of 2DG were enhanced and the cancer promoting effects of insulin were reversed. The glycolysis inhibition of 2DG was also promoted by insulin.

Western blot analysis for survival proteins, including Mcl-1 and survivin, was also analyzed. The result showed that 2DG inhibited the expressions of both proteins (Fig 4). Survivin is a member of the inhibitor of apoptosis (IAP) family. It is highly expressed in most human cancer cells. Survivin can inhibit caspase activation and suppress apoptosis.[27,28]. Mcl-1 is an anti-apoptotic member of the Bcl-2 family. It localizes to the mitochondria, antagonizes pro-apoptotic Bcl-2 family members, and inhibits apoptosis induced by cytotoxic stimuli. Mcl-1 is reported to be involved in cell death induced by inhibition of cell metabolism. Mcl-1 downregulation plays a critical role in 2DG induced apoptosis [7,29].

2DG administration at a low dose was not quite efficient to induce cell death and apoptosis. While, migration and invasion were markedly suppressed by 2DG, which was unlikely attributed to the modest cytotoxicity of 2DG at that dose. Sottnik et al. reported that 2DG was highly efficient to inhibit the metastatic phenotype of a large variety of tumor types both *in vitro* and *in vivo*, which was associated with cytoskeletal reorganization and inhibition of cathepsin L expression [30]. In this study, we detected the expression level of MMP2. Matrix metalloproteinases (MMPs) are a family of zinc- and calcium- dependent proteolytic enzymes that are involved in the breakdown of extracellular matrix and metastatic phenotype of cancers. As a key member of MMPs, MMP2 has been shown to be capable of degrading collagen IV, the major structural component of basement membrane [31–33].Western blot analysis showed that insulin promoted the expression of MMP2 protein, which might to some extent explain its promotion of invasion. In contrast, 2DG inhibited the expression of MMP2. Glycolysis was markedly inhibited by 2DG as demonstrated by the restricted productions of ATP and lactate. Due to ATP depletion and increased intracellular AMP/ATP ratio, AMPK, a key cellular energy sensor, is activated. AMPK activation attempts to recover ATP generation via switching on catabolism and switching off anabolism. ATP-consuming processes such as biosynthesis, cell growth and proliferation are restrained [34,35]. Cell cycle progression can be stalled at the G1-S phase transition [36]. Elevated glycolysis results in the generation of large quantities of lactate which must be transported out of cells in order to prevent poisoning themselves. Lactic acid transportation across the plasma membrane is mediated by a family of proton-linked monocarboxylate transporters (MCTs) [37]. Increased expression of MCT4 in tumor cells has been reported previously [38]. Our results showed that 2DG treatment led to decreased lactate production and downregulation of MCT4. The inhibitions of lactate production and extrusion are important aspects in the anticancer effects of 2DG since elevated lactate during glucose metabolism generally proves to be beneficial for cancer cells. On the one hand, lactate export results in the acidification of microenvironment which facilitates tumor angiogenesis and provides a favorable condition for the activation of cathepsins and metalloproteinases leading to extracellular matrix degradation and tumor cell metastasis [38,39]. On the other hand, extracellular acidification indirectly contributes resistance of cancer cells to radiation and some cytotoxic drugs, such as doxorubicin[40]. In addition, lactate is capable of inhibiting the differentiation of monocytes to dendritic cells and inactivating cytokine release thus contributing to immune escape of cancer cells [40]. Lactate may also favor adjacent aerobic tumor cell growth since it can be consumed to take the place of glucose for oxidative phosphorylation [38].

Based on many recent researches, the toxicity following 2DG treatment could be explained by more than one mechanism depending on the metabolic profile of a specific cancer cell type. Mimicking glucose deprivation, 2DG is capable of inducing autophagy [41–43]. The conversion from LC3-I to LC3-II is a representative process for autophagy flux [19]. A significantly upregulated expression of LC3 II protein was detected in 2DG treated colorectal cancer cells. Besides LC3, p62 is another maker of autophagy. p62 is a ubiquitin binding protein and is required for the formation of ubiquitinated protein aggregates. p62can be selectively incorporated into autophagosome through binding to LC3, leading the protein aggregates to degradation. Lysosomal degradation of autophagosomes leads to the decrease in p62 [19,44]. Western blot analysis showed decreased levels of p62 after 2DG administration. Although autophagy is commonly known as a cell survival mechanism under stressors, once extensively activated, the self-preservation may turn into massive destruction[45]. Despite the inhibition of glycolysis and induction of autophagy, 2DG can cause the alteration of N-linked glycosylation and intensification of oxidative stress [25,26,46].

Considering the side effects of 2DG, other highly glucose-dependent tissues despite cancer cells, such as brain, heart, retina and testes, are all potential victims [38]. Previous clinical trials confirmed that 2DG administration was generally safe and well tolerated by patients. Intriguingly, although intravenous administration of 2DG could cause hyperglycemia, side effects observed were quite similar to those of hypoglycemia [47–49].

As to the association between CRC and diabetes, meformin has also been found to be a potential drug against colorectal cancer through the regulation of AMPK and mammalian target of rapamycin (mTOR)—signaling pathway [50]. Similar to 2DG, meformin can inhibit the energy generation of cancer cell and affect cell metabolism. The combination of metformin and 2DG has been investigated to be much more effective in suppressing cancer cells through the induction of apoptosis. [51,52].

The strategy of combining 2DG and insulin proved to be promising against colorectal cancer and might be helpful in the treatment of other malignancies. One the one hand, insulin promoted 2DG uptake by cancer cells and increased the anticancer efficiency of 2DG. On the other hand, 2DG overcame the potential harmful effect of insulin. The mechanisms underlying the combinatory effects of insulin and 2DG are required to be further studied. Besides, *in vivo* researches about the administration of 2DG with and without insulin are needed to investigate not only the anticancer efficiency on colorectal cancer but also the effect on blood glucose level. To the best of our knowledge, no report has been issued on the concomitant administration of 2DG and insulin on colorectal cancer.

## Supporting Information

S1 Fig Date analysis of western blots. Western blot analysis of Phospho-AMPKα (Thr172), AMPKα, p62, LC3A/B, MMP2, SLC16A3/MCT4, Mcl-1, Survivin comparing with the control. *, P < 0.05. #, significant differences versus the controls (P < 0.05).
(TIF)
