# Supplementary figures and images for: 2-Deoxyglucose Reverses the Promoting Effect of Insulin on Colorectal Cancer Cells In Vitro
Source: PLoS One. 2016 Mar 3;11(3):e0151115. doi: 10.1371/journal.pone.0151115 (PMC4777557; doi:10.1371/journal.pone.0151115)

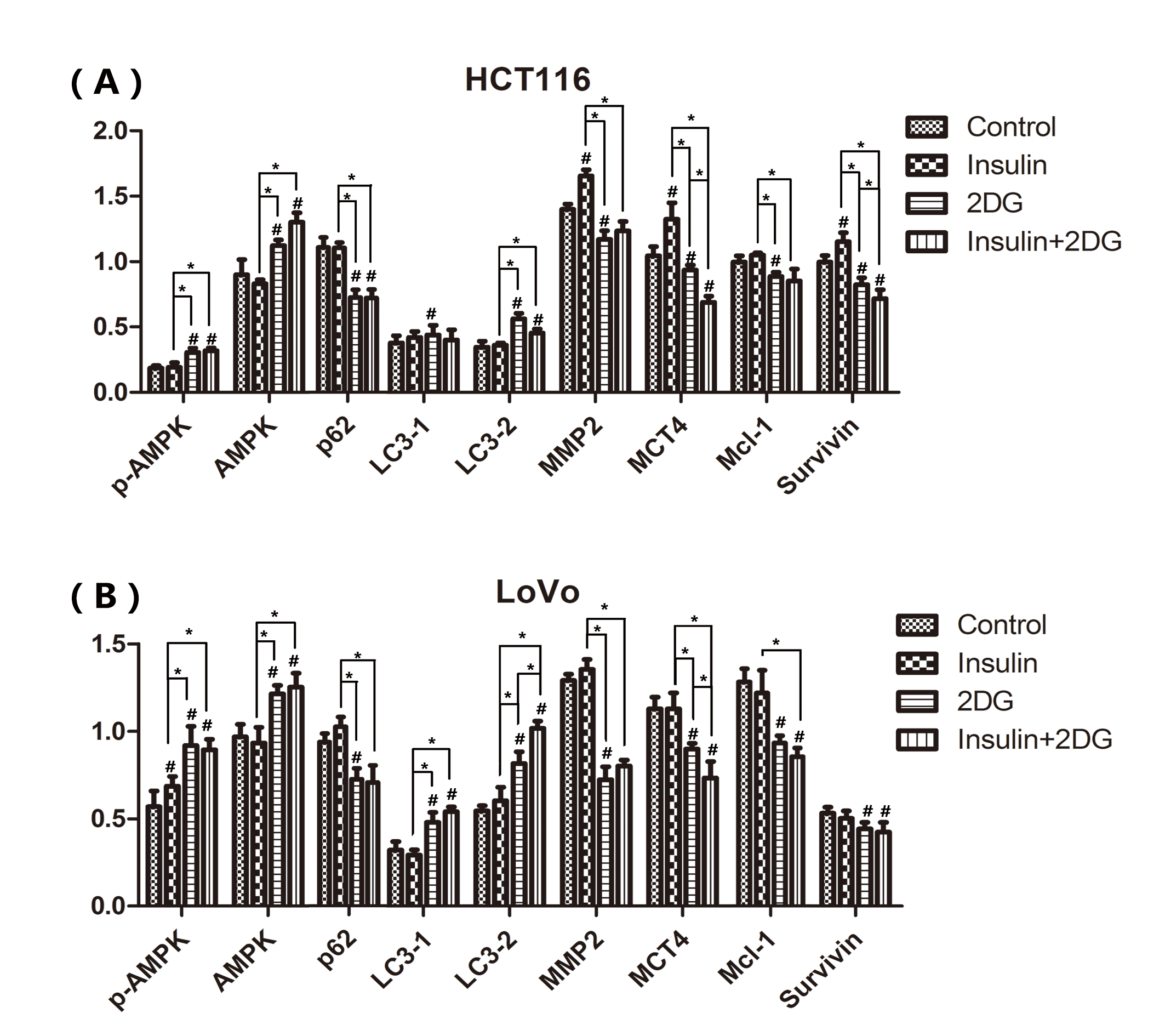

Supplement: S1 Fig — Western blot analysis of Phospho-AMPKα (Thr172), AMPKα, p62, LC3A/B, MMP2, SLC16A3/MCT4, Mcl-1, Survivin comparing with the control. *, P < 0.05. #, significant differences versus the controls (P < 0.05). (TIF) [file pone.0151115.s001.tif]
